# Identification of *Malassezia globosa* as a Gastric Fungus Associated with PD-L1 Expression and Overall Survival of Patients with Gastric Cancer

**DOI:** 10.1155/2022/2430759

**Published:** 2022-11-09

**Authors:** Zhenzhan Zhang, Yaopeng Qiu, Hao Feng, Donghua Huang, Binshu Weng, Zhou Xu, Qingfeng Xie, Zhe Wang, Wenfu Ding, Guoxin Li, Hao Liu

**Affiliations:** ^1^Department of General Surgery & Guangdong Provincial Key Laboratory of Precision Medicine for Gastrointestinal Tumor, Nanfang Hospital, Southern Medical University, Guangzhou, China; ^2^Department of Health Management, Nanfang Hospital, Southern Medical University, Guangzhou, China

## Abstract

**Background:**

Microbiotas affected the prognosis of cancer patients by regulating programmed death ligand-1 (PD-L1) expression. However, the relationship between gastric fungi and PD-L1 expression is still unclear in gastric cancer (GC). We aimed at exploring the association of gastric fungi with PD-L1 expression and overall survival in GC.

**Methods:**

A total of 61 GC patients were divided into the two groups based on the PD-L1 combined positive scores (CPS). Fungal profiling was performed by internal transcribed spacer rDNA sequencing, and the survival analyses were performed by Kaplan-Meier curves.

**Results:**

We observed a taxonomic difference of fungi between the PD-L1-High (*CPS* ≥ 10) and PD-L1-Low group (*CPS* < 10) by principal coordinates analysis (PCoA) (*P* = 0.014 for Bray-Curtis and *P* = 0.042 for Jaccard). *Malassezia* had a higher abundance in the PD-L1-High group compared to the PD-L1-Low group (*P* = 0.045). *Malassezia globosa* elevated significantly in the PD-L1-High group. GC patients with PD-L1 low expression and low abundance of *Malassezia globosa* had a longer overall survival (OS) than others (*P* = 0.047). *Malassezia globosa* was associated with PD-L1 expression (*Odds* *Ratio* = 3.509, 95% Confidence Interval: 1.056-11.656, *P* = 0.040). *Malassezia globosa* was associated with the tumor size (*P* = 0.031) and PD-L1 status (*P* = 0.024). GC patients with a high abundance of *Malassezia globosa* had shorter OS than others (*P* = 0.028). *Malassezia globosa* was an independent factor (*Hazard* *Ratio* = 3.080, 95% Confidence Interval: 1.140-8.323, *P* = 0.027) for OS after adjusting for tumor stage. *Malassezia globosa* was figured out to be associated with- fatty acid and lipid biosynthesis and degradation via LIPASYN pathway. *Conclusions. Malassezia globosa* was identified as a PD-L1 expression-associated gastric fungus and associated with OS of GC patients, which calls for more studies to further explore its potential in PD-L1/PD-1 targeted immunotherapy.

## 1. Introduction

Gastric cancer (GC) is the third leading cause of cancer-related death and an important global health problem [1]. Despite advances in chemotherapy and targeted therapy, the prognosis of GC was poor until the development of immunotherapy as a first-line treatment of advanced GC [2]. Programmed death ligand-1 (PD-L1) expression level is a predictor for the immunotherapeutic efficacy and outcomes of GC patients [3, 4]. An observational study reported that the use of antibiotics was associated with a poor prognosis in non-small-cell lung cancer (NSCLC) patients with PD-L1 expression ≥50% who received anti-PD-1 immunotherapy [5]. Microbiotas affect the survival prognosis of tumor patients who received immunotherapy depending on PD-L1 expression. Human gastrointestinal microbiotas are associated with PD-L1 expression in tumors. Gut microbiomes promote the polarization of M1/M2 macrophages, differentiation of Th1 lymphocytes, activation of cytotoxic T-cells, and upregulation of the expression of PD-L1 co-interactional molecule [6–8]. Intestinal microbiomes from the growth hormone-secreting pituitary adenoma (GHPA) patients promoted the mouse tumor growth, increased PD-L1 expression, and elevated the levels of PD-L1 in peripheral blood [9]. A great number of microbiotas including bacteria, fungi, and viruses colonize in the human stomach. Gastric microbiotas *Helicobacter pylori* (*H. pylori*) and Epstein-Barr virus are associated with PD-L1 expression. *H. pylori* induces early gastric epithelial cell response to increase PD-L1 expression [10], whereas *H. pylori* mediates SOCS3 signaling to inhibit PD-L1 expression in human monocyte-derived dendritic cells from patients with *H. pylori*-positive gastritis [11]. *H. pylori*-positive gastric intraepithelial neoplasia (GIN) and early-stage GC are associated with PD-L1 expression [12]. The *H. pylori*-derived virulence factors, CagA and VacA affect PD-L1 expression in the development of gastric immunopathology [13]. Moreover, PD-L1 overexpression is a unique characteristic in Epstein-Barr virus-positive GC [14]. Epstein-Barr virus induces host miR-BART5-5p to upregulate the expression of PD-L1 [15]. EBV nuclear antigen-1 (EBNA1) promotes the IFN-*γ*-induced PD-L1 expression [16]. However, the relationship between other gastric microbiotas and PD-L1 expression is unclear in cancers. Given that fungi are one of major components of gastric microbiotas, the relationships between gastric fungi and PD-L1 expression in GC need further exploration.

In this study, 61 GC patients without any preoperative treatments were enrolled retrospectively. We aimed at exploring the role of gastric fungi in PD-L1 expression and prognosis for GC, providing a better understanding of PD-L1 expression-associated gastric fungi and their association with prognosis, and helping- identify the novel antifungal targets for therapeutic interventions in GC.

## 2. Methods

### 2.1. Study Population and Specimen Collection

In total, 61 GC patients without preoperative chemotherapy, radiotherapy, and immunotherapy were enrolled from March 2020 to September 2020 at Nanfang Hospital, Southern Medical University (Guangdong, China). These patients did not receive antibiotics, proton pump inhibitors, or H2 receptor antagonists within 1 month. All patients were provided the informed consent for collection of tumor tissues after they underwent gastrectomy. The patient characteristics are shown in Table 1. The overall survival (OS) and disease-free survival (DFS) time were described by Kaplan-Meier curves. The tumor tissues were collected under aseptic conditions and immediately stored at -80°C.

### 2.2. Immunohistochemistry for PD-L1

The tumor tissues were sent for Hematoxylin-Eosin (H-E) staining for pathological diagnosis. PD-L1 expression was evaluated by immunohistochemistry (IHC) staining. IHC of PD-L1 protein was carried out on a Dako Autostainer Link 48 system (Dako, Carpenteria, CA) by using the PD-L1 IHC kit (Dako, PD-L1, 22C3, pharmDx, Agilent Technologies) with the Envision FLEX visualization system according to the manufacturer's instructions. The combined positive score (CPS) was calculated as the number of PD-L1-positive cells (tumor cells, lymphocytes, macrophages) divided by the number of all viable tumor cells and then multiplied by 100 to define PD-L1 expression. PD-L1 positivity was defined as *CPS* ≥ 1, and cell counts and CPS categorization were performed by experienced pathologists [17]. In this study, PD-L1 positivity *CPS* ≥ 10 was defined as high PD-L1 expression, and PD-L1 positivity *CPS* < 10 was defined as low PD-L1 expression according to previous studies [18].

### 2.3. Sequencing Procedure

Paired-end sequencing was performed on a NovaSeqPE250 Platform (Illumina, San Diego, CA, U.S.A.). The ITS1-5F regions of ITS rDNA gene were amplified by primers as previously described [19]. The primer sequences were ITS5-1737F: 5′- GGAAGTAAAAGTC GTAACAAGG-3′ and ITS2-2043R: 5′-GCTGCGTTCTTCATC GATGC-3′. The raw data were filtered with QIIME and DADA2 methods, clustered into operational taxonomic units (OTUs) or amplicon sequence variants (ASVs) with at least 97% similarity. The classification of ASVs or OTUs was performed by the UNITE database (Release 8.0).

### 2.4. Data Analysis

Alpha-diversity analysis was used to reflect the richness of microbiotas. The rank abundance curves and species accumulation curves were used to describe the relative abundance of ASVs or OTUs. Principal coordinates analysis (PCoA) was performed to analyze the difference. The analysis of similarity (ANOSIM) and Adonis was used to test the dissimilarity between the groups. A Venn diagram was used to indicate the distribution. Linear discriminant analysis (LDA) effect size (LEfSe) was applied to find biomarkers, and differences with LDA scores greater than 2 and *P* value < 0.05 were considered significant. The random-forest-classifier was used to evaluate the contribution in grouping differences. The Phylogenetic Investigation of Communities by Reconstruction of Unobserved States (PICRUSt, verson.2) was applied for functional prediction. Statistical data were calculated by using SPSS V22.0 (Chicago, IL, U.S.A.), Welch's *t* test for the continuous variables analysis, and Pearson's chi-square test for the categorical variable analysis. *P* value < 0.05 was considered statistically significant.

## 3. Results

### 3.1. A Taxonomic Difference of Gastric Fungi between the PD-L1-High and PD-L1-Low Group

**Figure 1:**
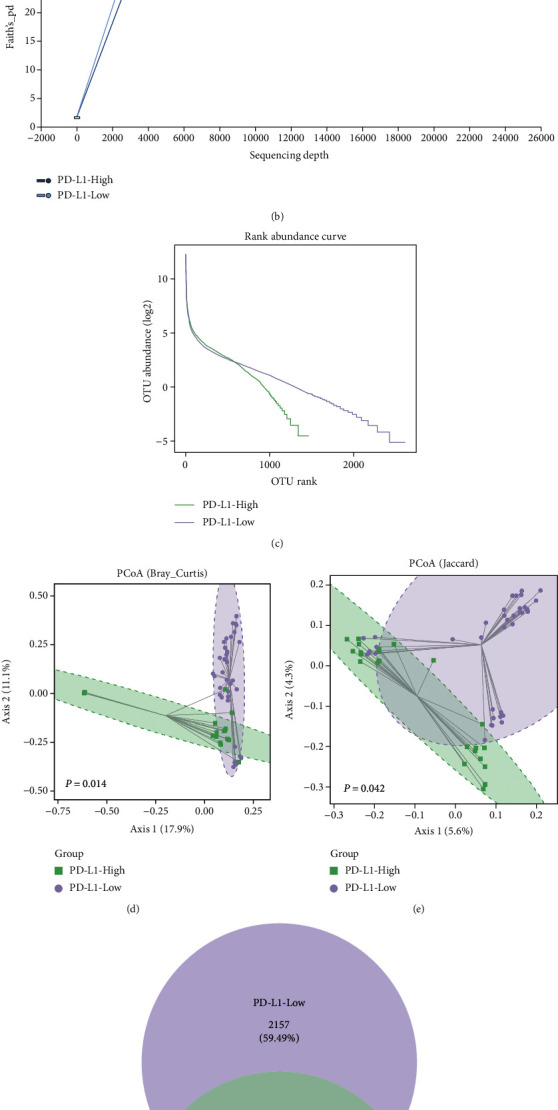
A taxonomic difference of fungi in the different PD-L1 expression groups. (a) H-E and IHC staining for PD-L1 expression. (b and c) The alpha-rarefaction curves and rank abundance curves. (d and e) Based on the Bray-Curtis and Jaccard distance, PCoA in two groups (ANOSIM and Adonis test). (f) The Venn diagram.

These 61 GC patients were divided into two groups according to the IHC density of PD-L1 expression as previously described. Based on a defined PD-L1 expression level of *CPS* = 10, 24 patients were included in the PD-L1-High group (*CPS* ≥ 10) and 37 patients were included in the PD-L1-Low group (*CPS* < 10) (Figure 1(a)). Clinical characteristics analysis indicated that the two groups had no significant differences (Supplementary Table 1). The tumor tissues from two groups were collected for ITS rDNA sequencing. The alpha-rarefaction curves and rank abundance curves showed that a lower abundance was found in the PD-L1-High group than in the PD-L1-Low group (Figures 1(b) and 1(c)). Alpha-diversity analysis showed the negative differences between the two groups (Supplementary Figure 1). However, PCoA indicated that the PD-L1-High and PD-L1-Low groups aggregated separately (*P* = 0.014 for Bray-Curtis and *P* = 0.042 for Jaccard) (Figures 1(d) and 1(e)). A Venn diagram showed the overlapping section of OTU and demonstrated a lower abundance of OTU in the PD-L1-High group than the PD-L1-Low group (Figure 1(f)). Taken together, the results suggested a taxonomic difference of fungi between the PD-L1-High and PD-L1-Low group.

### 3.2. *Malassezia* Elevated Significantly in the PD-L1-High Group

**Figure 2:**
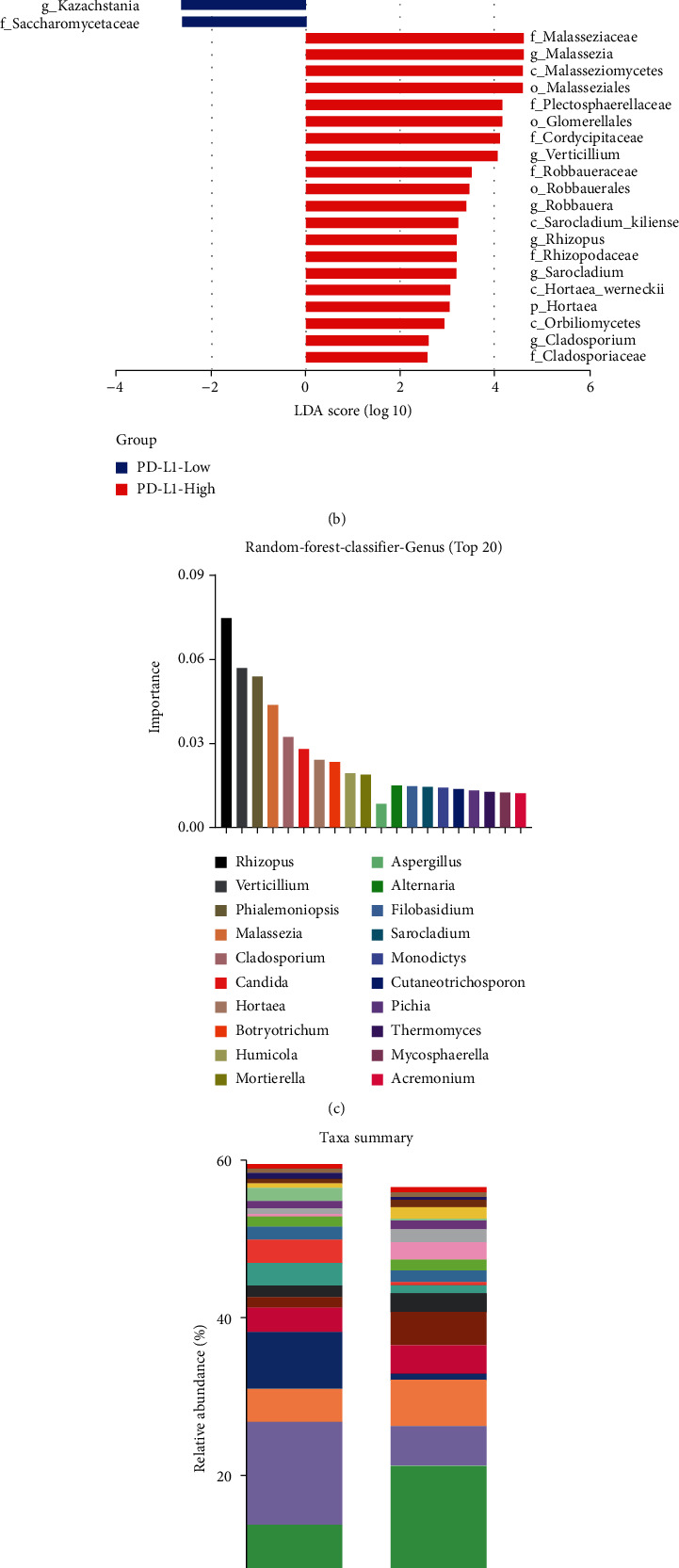
The fungal microbiota composition in the different PD-L1 expression groups. (a) Cladogram representation between the PD-L1-High and PD-L1-Low group. (b) Histogram of the linear discriminant analysis (LDA) scores. (c) The random-forest-classifier analysis. (d) The taxa summary analysis. (e) The Venn diagram. (f) Welch's *t* test was used to analyze between the two groups.

To further evaluate the taxa difference between the PD-L1-High and PD-L1-Low group, we performed the discriminant analyses using LEfSe, which showed that 27 fungal phylotypes were significantly different between the two groups: 7 fungal phylotypes differentially enriched in the PD-L1-Low group and 20 fungal phylotypes differentially enriched in the PD-L1-High group (Figures 2(a) and 2(b)). We used the random-forest-classifier to identify the importance of top 20 fungal microbiomes in grouping differences at the genus level (Figure 2(c)). The taxa summary indicated the relative abundance of top 20 fungal microbiomes between the two groups at the genus level (Figure 2(d)). After a comprehensive analysis of the above results, *Malassezia* and *Verticillium* were identified out (Figure 2(e)). Moreover, the abundance of *Malassezia* and *Verticillium* was evaluated based on the Welch's *t* test, the results of which showed that *Malassezia* had higher abundance and was significantly elevated in the PD-L1-High group compared to the PD-L1-Low group (*P* = 0.045) (Figure 2(f)). Finally, *Malassezia* was chosen for analysis between the PD-L1-High and PD-L1-Low group.

### 3.3. *Malassezia Globosa* Was Associated with PD-L1 Expression in GC

**Figure 3:**
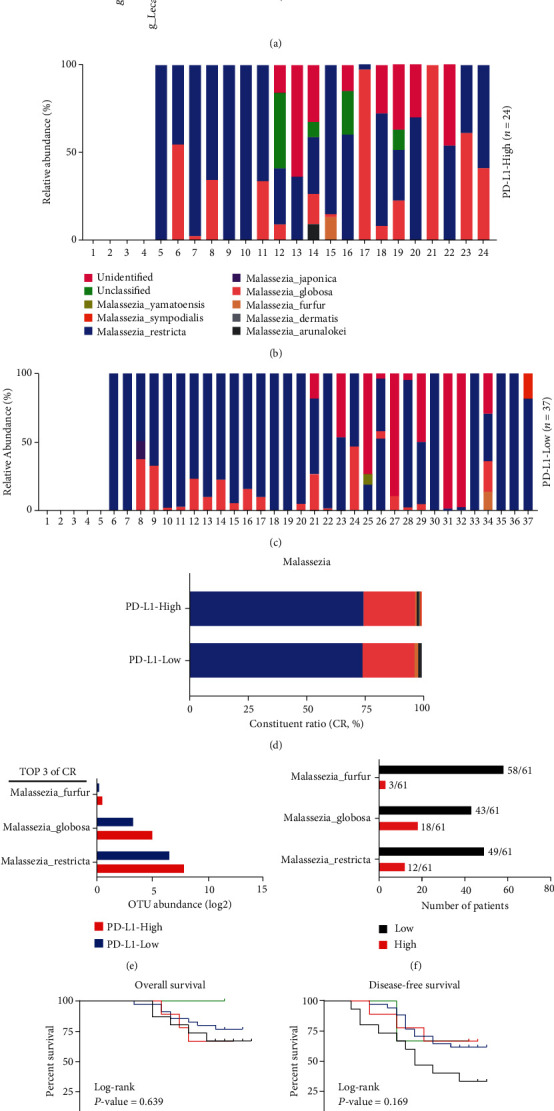
*Malassezia globosa* was a PD-L1 expression-associated gastric fungus. (a) The distribution of *Malassezia.* (b and c) The species of *Malassezia* in the PD-L1-High and PD-L1-Low group. (d) Constituent ratio analysis. (e) The OTU abundance of *Malassezia globosa, Malassezia restricta*, and *Malassezia furfur* between the two groups. (f) The high abundance group and low abundance group based on the OTU abundance of *Malassezia restricta* (cutoff value = 1238), *Malassezia globosa* (cutoff value = 140) and *Malassezia furfur* (cutoff value = 3). (g) Survival analysis in the different subgroups of *Malassezia restricta* and PD-L1 expression. (h) The OS and DFS time in the different subgroups of *Malassezia globosa* and PD-L1 expression.

To have a better understanding of *Malassezia*, a krona pie chart was used to describe the distribution of *Malassezia* in GC. *Malassezia* accounted for 11% of gastric fungi in the GC microenvironment (Figure 3(a)). At the species level, the relative abundance of the fungal species of *Malassezia* was described in the PD-L1-High and PD-L1-Low group, respectively (Figures 3(b) and 3(c)). We observed that *Malassezia restricta*, *Malassezia globosa,* and *Malassezia furfur* were the main species of *Malassezia* in the two groups (Figure 3(d)). Three fungi were elevated in the PD-L1-High group compared to the PD-L1-Low groups (Figure 3(e)). Based on the OTU abundance of *Malassezia restricta*, *Malassezia globosa,* and *Malassezia furfur*, 61 GC patients were divided into high abundance group and low abundance group, respectively (Figure 3(f), Supplementary Table 2). Survival analysis indicated that the OTU abundance of *Malassezia restricta* was not associated with the prognosis of GC patients (*P* = 0.639 for overall survival (OS) and *P* = 0.169 for disease-free survival (DFS)) (Figure 3(g)). However, a subgroup including GC patients with both PD-L1 low expression (*CPS* < 10) and low abundance of *Malassezia globosa* (OTU *abundance* < 140) had a better prognosis than other subgroups (*P* = 0.047 for OS and *P* = 0.249 for DFS) (Figure 3(h)). *Malassezia globosa* was further analyzed the association with PD-L1 expression. Multivariate analysis of logistic regression indicated that the abundance of *Malassezia globosa* (OTU *abundance* ≥ 140) was associated with the expression of PD-L1 (*Odds* *Ratio* = 3.509, 95% Confidence Interval: 1.056-11.656, *P* = 0.040) (Table 2). Clinical characteristic analysis showed that *Malassezia globosa* was associated with the tumor size (*P* = 0.031) and PD-L1 status (*P* = 0.024), respectively (Supplementary Table 3).

Interestingly, compared to *Malassezia restricta* and *Malassezia furfur*, *Malassezia globosa* was associated with the outcomes of GC patients. These GC patients with a high abundance of *Malassezia globosa* had shorter OS than other patients with a low abundance of *Malassezia globosa* (*P* = 0.028) (Supplementary Figure 2). Cox regression analysis revealed that *Malassezia globosa* was an independent factor (Hazard Ratio = 3.080, 95% Confidence Interval: 1.140-8.323, *P* = 0.027) for OS after adjustment of tumor stage (Supplementary Table 4). Therefore, *Malassezia globosa* may be a PD-L1 expression-associated gastric fungus and be associated with OS of GC patients.

### 3.4. Functional Prediction of *Malassezia Globosa* and PD-L1 Expression

**Figure 4:**
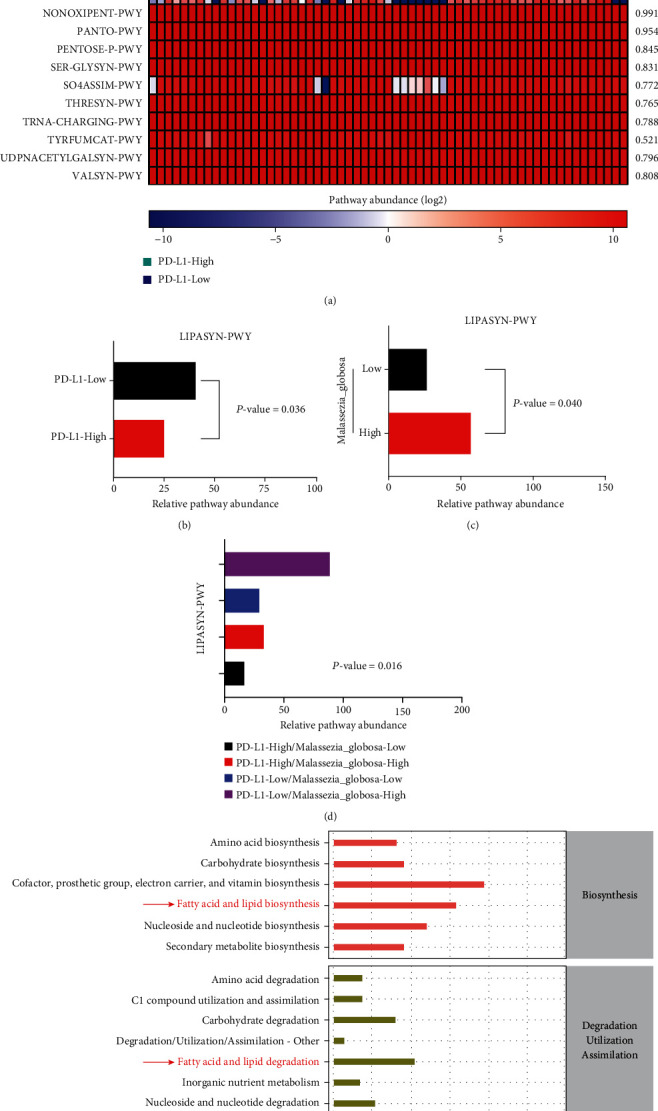
Prediction of pathway related to *Malassezia globosa* and PD-L1 expression. (a) The enrichment of metabolic pathways between the PD-L1-High and PD-L1-Low group. (b) The association between PD-L1 expression and LIPASYN pathway. (c) The association between *Malassezia globosa* and LIPASYN pathway. (d) The enrichment of LIPASYN pathway in the different subgroups of *Malassezia globosa* and PD-L1 expression. (e) The metabolism-related processes were enriched.

The PICRUSt.2 was used to predict the function of *Malassezia globosa* between the PD-L1-High and PD-L1-Low group. Based on the MetaCyc database, the abundance of metabolic pathways was evaluated. The results showed that LIPASYN-PWY was predicted to be a different metabolic pathway between the PD-L1-High and PD-L1-Low group (*P* = 0.036) (Figures 4(a) and 4(b)). Meanwhile, *Malassezia globosa* was also predicted to be associated with LIPASYN-PWY (*P* = 0.040) (Figure 4(c)). Most importantly, the enrichment in the four subgroups of *Malassezia globosa* and PD-L1 expression suggested that LIPASYN-PWY was a significantly different pathway among the four subgroups (*P* = 0.016) (Figure 4(d)). LIPASYN-PWY is related to fatty acid and lipid biosynthesis and degradation. These results were consistent with the fact that the pathway abundance of gastric fungi was mainly enriched in metabolic process of fatty acid and lipid biosynthesis and degradation (Figure 4(e)). Functional prediction implied that *Malassezia globosa* was associated with the process of fatty acid and lipid biosynthesis and degradation via LIPASYN pathway between the PD-L1-High group and PD-L1-Low group.

## 4. Discussion

In this study, we demonstrated a taxonomic difference of gastric fungi between the PD-L1-High and PD-L1-Low group via ITS rDNA sequencing for the first time, and further revealed that *Malassezia globosa* was associated with PD-L1 expression in the GC microenvironment. Moreover, *Malassezia globosa* was considered to be a predictive gastric fungus for the prognosis of GC patients.

*Malassezia* is a lipid-dependent opportunistic yeast that colonizes in human skin, mucosa, and other warm-blooded animals. *Malassezia* causes several skin diseases including pityriasis versicolor, seborrheic dermatitis, folliculitis, and dandruff [20]. *Malassezia* has at least 14 species, eight of which have been isolated from human skin, including *Malassezia japonica*, *Malassezia yamatoensis*, *Malassezia furfur*, *Malassezia sympodialis*, *Malassezia arunalokei*, *Malassezia globosa*, *Malassezia restricta,* and *Malassezia dermatis* [21]. With the development of high-throughput sequencing, *Malassezia* does not limit to human skin only, but also is a resident fungus in different parts of human body such as the oral cavity, gastrointestinal tract, respiratory tract, brain, and breast milk [22]. Many studies about the *Malassezia* in human gastrointestinal tract have emerged in recent years. The detection rate of *Malassezia* in the digestive tract of healthy individuals is up to 81-88.3%, and the rate of diseased individuals is also up to 68.4-100%, respectively [23–26].

*Malassezia* is known to be a human skin-colonized mycobiota, but the high detection rate of *Malassezia* in the human digestive tract is controversial. Most studies reported that *Malassezia* was derived from human intestines, while other studies revealed the *Malassezi*a also existed in human stomach. Our study provided evidence that *Malassezia* was present in the stomach environment in GC patients by ITS rDNA sequencing. At the species level, based on the OTU abundance, *Malassezia globosa* was presented and identified in the gastric tumor tissues. We further demonstrated the clinical relevance and prognosis of GC patients with the presence of *Malassezia globosa*. We found *Malassezia globosa* was an independent factor for OS after adjusting for tumor stage. The presence of *Malassezia globosa*in human stomach may play an important role in GC patients.

As a common pathogenic fungus, *Malassezia* has been reported to be associated with inflammation and cancers. *Malassezia* induces inflammatory immune response and tumorigenesis. *Malassezia* induces IL-17-dependent inflammation and mediated fungal infection via keratinocyte IL-36 receptor/MyD88 signaling in mouse skin. As the species of *Malassezia*, *Malassezia globosa* and *Malassezia restricta* can induce proinflammatory cytokine IL-1*β* production and activate the NLRP3 inflammasome in phagocytes [27]. *Malassezia* is a crucial microbial factor in carcinogenesis, which is an accomplice of evoking tumorigenesis for promoting the progression of inflammatory bowel disease and contributes to a worse prognosis [28].

*Malassezia* has a higher abundance in colorectal cancer (CRC) compared to the colon polyp. Gut-derived *Malassezia globosa* markedly enriches in both mice and human pancreatic ductal adenocarcinoma cancer (PDAC) and promotes pancreatic oncogenesis by driving the complement cascade through the activation of MBL [29, 30]. In addition to the *Malassezia*-associated CRC and PDAC, we focused on GC and immune-suppressive checkpoint, revealing the association of *Malassezia globosa* and PD-L1 expression in the GC microenvironment. *Malassezia globosa* was an independent factor for PD-L1 expression after adjusting for other clinical characteristics in GC. *Malassezia globosa* was a predictor for OS after adjustment for tumor stage. *Malassezia globosa* may be a predictive gastric fungus for the outcomes of GC patients with PD-L1 expression. As a lipid-dependent fungus, *Malassezia globosa* is associated with the metabolic process of fatty acid and lipid biosynthesis and degradation via LIPASYN pathway between the PD-L1-High group and PD-L1-Low group. *Malassezia globosa* may have association with PD-L1 expression in GC via lipid metabolism. However, the mechanism between *Malassezia globosa* and PD-L1 expression is unclear. We need more evidences to understand whether the high abundance of *Malassezia globosa* can promote high expression of PD-L1 or high expression of PD-L1 and further induces the high abundance of *Malassezia globosa* in the GC microenvironment. Besides, the lipid metabolites from *Malassezia globosa* in GC need to be further analyzed by metabolomics.

In summary, our study first provided novel insights into*Malassezia globosa* and PD-L1 expression in the GC microenvironment. *Malassezia globosa* has the potential to be a PD-L1 expression-associated gastric fungus and a predictive biomarker for OS of GC patients. The combined effect of antifungal therapy targeting *Malassezia globosa* and antiPD1/PD-L1 immunotherapy on GC should be investigated in the future.

## Figures and Tables

**Table 1:** Baseline characteristics of the study subjects (*n* = 61).

Characteristics	Median (range)/number (%)
Age (years)	56 (22-86)
Gender	
Male	43 (70.5%)
Female	18 (29.5%)
Body mass index (kg/m^2^)	22.1 (16.6-35.1)
Tumor size (max, cm) [median (range)]	4.6 (0.5-15)
Tumor location	
Upper	19 (31.1%)
Middle	12 (19.7%)
Lower	30 (49.2%)
Tumor differentiation	
High	4 (6.5%)
Moderate	19 (31.1%)
Poor	38 (62.3%)
Lauren classification	
Diffuse	36 (59.0%)
Intestinal	17 (27.9%)
Mix	8 (13.1%)
pTNM stage	
I	6 (9.8%)
II	9 (14.8%)
III	34 (55.7%)
IV	12 (19.7%)
Lymphatic vessel invasive (D2-40)	
Positive	20 (32.8%)
Negative	41 (67.2%)
Vascular invasive (CD31)	
Positive	15 (24.6%)
Negative	46 (75.4%)
Nerve invasive (S-100)	
Positive	46 (75.4%)
Negative	15 (24.6%)
HER2 status	
0/1+/2+	53 (86.9%)
3+	8 (13.1%)
PD-L1 status	
CPS ≥ 10	24 (39.3%)
CPS < 10	37 (60.7%)
Postoperative treatment	
None	6 (9.8%)
Chemotherapy	41 (67.2%)
Chemotherapy+anti-PD-1 immunotherapy	14 (23.0%)

**Table 2:** The association of clinical characteristics with expression level of PD-L1.

Characteristics	Univariate analysis^#^		Multivariate analysis	
	OR, 95% CI	*P* value	OR, 95% CI	*P* value
Age (≥ 65 vs. < 65, years)	1.763 (0.612-5.076)	0.293	**—**	
Gender (male vs. female)	2.058 (0.624-6.794)	0.236	**—**	
BMI (≥ 24 vs. < 24, kg/m^2^)	1.556 (0.501-4.831)	0.445	**—**	
Tumor max size (≥ 4.6 vs. < 4.6, cm)	2.187 (0.764-6.261)	0.145	**—**	
Tumor location				
Upper	1 (ref.)			
Middle	0.300 (0.061-1.467)	0.137	**—**	
Lower	0.521 (0.162-1.674)	0.274	**—**	
Tumor differentiation (poor vs. high/moderate)	1.014 (0.351-2.929)	0.979	**—**	
Lauren classification				
Intestinal	1 (ref.)			
Diffuse	0.807 (0.248-2.631)	0.723	**—**	
Mix	1.429 (0.264-7.737)	0.679	**—**	
pTNM stage (IV vs. I/II/III)	0.444 (0.107-1.846)	0.264	**—**	
Lymphatic vessel invasive (positive vs. negative)	2.632 (0.877-7.904)	0.084	1.651 (0.469-5.806)	0.435
Vascular invasive (positive vs. negative)	3.100 (0.931-10.323)	0.065	2.595 (0.662-10.168)	0.171
Nerve invasive (positive vs. negative)	0.976 (0.894-1.065)	0.584	**—**	
HER2 status (3+ vs. 0/1+/2+)	1.650 (0.371-7.344)	0.511	**—**	
*Malassezia globosa* (high vs. low)	3.626 (1.149-11.448)	0.028	3.509 (1.056-11.656)	0.040

^**#**^The variables with *P* < 0.1 were included in multivariate analysis. ^∗^*P* < 0.05 was considered significant. OR: odds ratio; CI: confidence interval.

## Data Availability

All raw data were deposited into the NCBI SRA database (https://www.ncbi.nlm.nih.gov/sra/, accession number: SUB10789578 and Bioproject PRJNA812999).

## Supplementary Materials

Supplementary 1 Supplementary Figure 1: alpha-diversity of the gastric fungal microbiota in the different PD-L1 expression groups.

Supplementary 2 Supplementary Figure 2: (A-C) survival analyses of *Malassezia globosa*, *Malassezia restricta*, and *Malassezia furfur* in GC patients.

Supplementary 3 Supplementary Table 1: clinical characteristics of 61 GC patients divided into two groups according to the IHC density of PD-L1 expression in tumor tissues.

Supplementary 4 Supplementary Table 2: the fungal species of *Malassezia* at the species level.

Supplementary 5 Supplementary Table 3: clinicopathological characteristics of *Malassezia globosa* in GC patients.

Supplementary 6 Supplementary Table 4: univariate and multivariable Cox regression analyses for OS.
