# Supplementary material for: Identification of Malassezia globosa as a Gastric Fungus Associated with PD-L1 Expression and Overall Survival of Patients with Gastric Cancer
Source: J Immunol Res. 2022 Nov 9;2022:2430759. doi: 10.1155/2022/2430759 (PMC9669766; doi:10.1155/2022/2430759)

Supplementary Figure.1 Figure Legend

Alpha-diversity of the gastric fungal microbiota in the different PD-L1 expression groups.

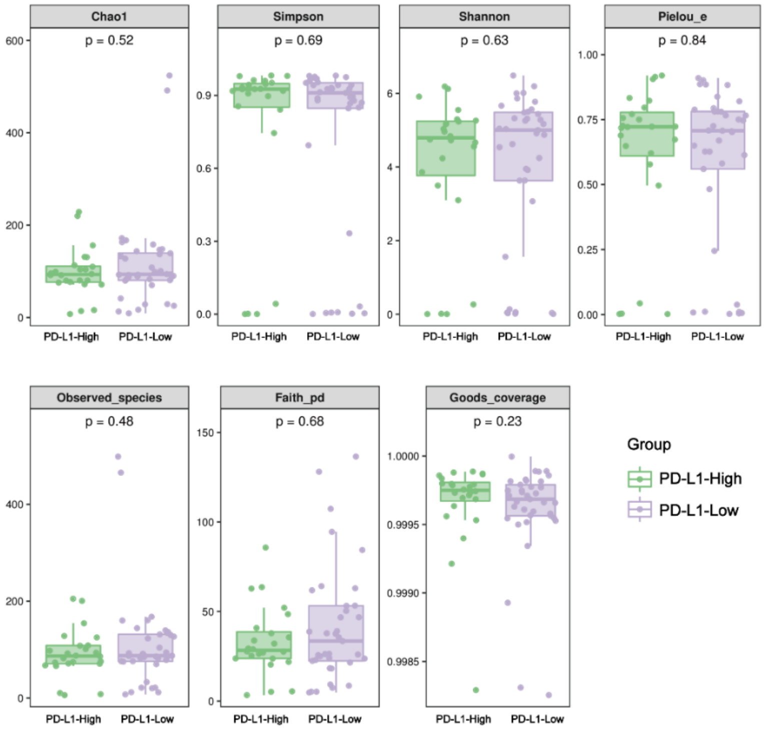

Supplement: Supplementary 1 — Supplementary Figure 1: alpha-diversity of the gastric fungal microbiota in the different PD-L1 expression groups. [file 2430759.f1.pdf]
