# Supplementary material for: Identification of Malassezia globosa as a Gastric Fungus Associated with PD-L1 Expression and Overall Survival of Patients with Gastric Cancer
Source: J Immunol Res. 2022 Nov 9;2022:2430759. doi: 10.1155/2022/2430759 (PMC9669766; doi:10.1155/2022/2430759)

Supplementary Figure.2 Figure Legend

(A-C) Survival analysis of *Malassezia globosa*, *Malassezia restricta*, *Malassezia furfur* in GC patients

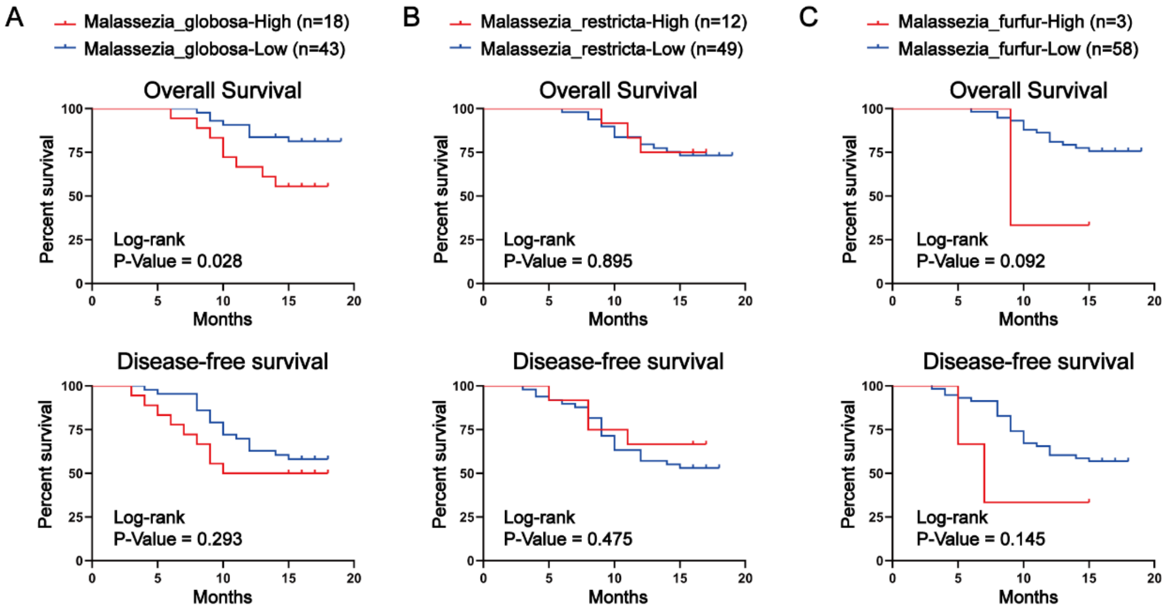

Supplement: Supplementary 2 — Supplementary Figure 2: (A-C) survival analyses of Malassezia globosa, Malassezia restricta, and Malassezia furfur in GC patients. [file 2430759.f2.pdf]
