# Supplementary material for: Identification of Malassezia globosa as a Gastric Fungus Associated with PD-L1 Expression and Overall Survival of Patients with Gastric Cancer
Source: J Immunol Res. 2022 Nov 9;2022:2430759. doi: 10.1155/2022/2430759 (PMC9669766; doi:10.1155/2022/2430759)
Supplement: Supplementary 3 — Supplementary Table 1: clinical characteristics of 61 GC patients divided into two groups according to the IHC density of PD-L1 expression in tumor tissues. [file 2430759.f3.pdf]

Supplementary.Table.1 Clinical characteristics of 61 GC patients who divided into the two groups according to the IHC density of PD-L1 expression in tumor tissues.

Characteristics		PD-L1 expression (CPS Index)		
		High (n=24)	Low (n=37)	P-value
Age	≥ 65	11 (45.8%)	12 (32.4%)	
	< 65	13 (54.2%)	25 (67.6%)	0.291
Gender	Male	19 (79.2%)	24 (64.9%)	
	Female	5 (20.8%)	13 (35.1%)	0.232
Body Mass Index (kg/m2)	< 24	16 (66.7%)	28 (75.7%)	
	≥ 24	8 (33.3%)	9 (24.3%)	0.443
Tumor Location	Upper	10 (41.7%)	9 (24.3%)	
	Middle/Lower	14 (58.3%)	28 (75.7%)	0.153
Tumor Differentiation	High/Moderate	9 (37.5%)	14 (37.8%)	
	Poor	15 (62.5%)	23 (62.2%)	0.979
Lauren Classification	Diffuse	13 (54.2%)	23 (62.2%)	
	Intestinal	7 (29.1%)	10 (27.0%)	
	Mix	4 (16.7%)	4 (10.8%)	0.755
Tumor Size (Max, cm)	≥ 4.6	15 (62.5%)	16 (43.2%)	
	< 4.6	9 (37.5%)	21 (56.8%)	0.142
pTNM Stage	I	2 (8.3%)	4 (10.8%)	
	II	2 (8.3%)	7 (18.9%)	
	III	17 (70.9%)	17 (46.0%)	
	IV	3 (12.5%)	9 (24.3%)	0.278
Lymphatic Vessel Invasive (D2-40)	Positive	11 (45.8%)	9 (24.3%)	
	Negative	13 (54.2%)	28 (75.7%)	0.080
Vascular Invasive (CD31)	Positive	9 (37.5%)	6 (16.2%)	
	Negative	15 (62.5%)	31 (83.9%)	0.059
Nerve Invasive (S-100)	Positive	19 (79.2%)	27 (73.0%)	
	Negative	5 (20.8%)	10 (27.0%)	0.583
HER2 Status	0/1+/2+	20 (83.3%)	33 (89.2%)	
	3+	4 (16.7%)	4 (10.8%)	0.508
Postoperative Treatment	None	2 (8.3%)	4 (10.8%)	
	Chemotherapy	14 (58.3%)	27 (73.0%)	
	Chemotherapy+Anti-PD-1 Immunotherapy	8 (33.3%)	6 (16.2%)	0.299

*P<0.05 was considered significant. Chi-square test was used in this table.
