# Supplementary material for: Identification of Malassezia globosa as a Gastric Fungus Associated with PD-L1 Expression and Overall Survival of Patients with Gastric Cancer
Source: J Immunol Res. 2022 Nov 9;2022:2430759. doi: 10.1155/2022/2430759 (PMC9669766; doi:10.1155/2022/2430759)
Supplement: Supplementary 4 — Supplementary Table 2: the fungal species of Malassezia at the species level. [file 2430759.f4.pdf]

Supplementary.Table.2 The fungal species of *Malassezia* at the species level.

Malassezia	AUC，95%CI	Cutoff value	OR, 95%CI	P-value
Malassezia_globosa	0.614 (0.465, 0.762)	140	3.63 (1.17-11.96)	0.028
Malassezia_restricta	0.624 (0.469, 0.778)	1238	6.80 (1.75-34.06)	0.009
Malassezia_furfur	0.528 (0.466, 0.591)	3	3.27 (0.30-72.89)	0.345
Malassezia_arunalokei	0.508 (0.459, 0.557)	591	-	-
Malassezia_dermatis	-	-	-	-
Malassezia_japonica	-	-	-	-
Malassezia_sympodialis	-	-	-	-
Malassezia_yamatoensis	-	-	-	-
