# Supplementary material for: Identification of Malassezia globosa as a Gastric Fungus Associated with PD-L1 Expression and Overall Survival of Patients with Gastric Cancer
Source: J Immunol Res. 2022 Nov 9;2022:2430759. doi: 10.1155/2022/2430759 (PMC9669766; doi:10.1155/2022/2430759)
Supplement: Supplementary 5 — Supplementary Table 3: clinicopathological characteristics of Malassezia globosa in GC patients. [file 2430759.f5.pdf]

Supplementary.Table.3 Clinicopathological characteristics of *Malassezia globosa* in GC patients.

Characteristics		*Malassezia globosa*		
		Low (n=43)	High (n=18)	P-value
Age	≥ 65	13 (30.2%)	10 (55.6%)	
	< 65	30 (69.8%)	8 (44.4%)	0.063
Gender	Male	31 (72.1%)	12 (66.7%)	
	Female	12 (27.9%)	6 (33.3%)	0.672
Body Mass Index (kg/m2)	< 24	31 (72.1%)	13 (72.2%)	
	≥ 24	12 (27.9%)	5 (27.8%)	0.992
Tumor Location	Upper	11 (25.6%)	8 (44.4%)	
	Middle/Lower	32 (74.4%)	10 (55.6%)	0.147
Tumor Differentiation	High/Moderate	19 (44.2%)	4 (22.2%)	
	Poor	24 (55.8%)	14 (77.8%)	0.106
Lauren Classification	Diffuse	24 (55.8%)	12 (66.7%)	
	Intestinal	13 (30.2%)	4 (22.2%)	
	Mix	6 (14.0%)	2 (11.1%)	0.732
Tumor Size (Max, cm)	≥ 4.6	18 (41.9%)	13 (72.2%)	
	< 4.6	25 (58.1%)	5 (27.8%)	**0.031***
pTNM Stage	I	4 (9.3%)	2 (11.1%)	
	II	9 (20.9%)	0 (0%)	
	III	22 (51.2%)	12 (66.7%)	
	IV	8 (18.6%)	4 (22.2%)	0.218
Lymphatic Vessel Invasive (D2-40)	Positive	12 (27.9%)	8 (44.4%)	
	Negative	31 (72.1%)	10 (55.6%)	0.210
Vascular Invasive (CD31)	Positive	10 (23.3%)	5 (27.8%)	
	Negative	33 (76.7%)	13 (72.2%)	0.708
Nerve Invasive (S-100)	Positive	31 (72.1%)	15 (83.3%)	
	Negative	12 (27.9%)	3 (16.7%)	0.352
HER2 Status	0/1+/2+	35 (81.4%)	18 (100%)	
	3+	8 (18.6%)	0 (0%)	0.050
PD-L1 Status	CPS≥10	13 (30.2%)	11 (61.1%)	
	CPS<10	30 (69.8%)	7 (38.9%)	**0.024***
Postoperative Treatment	None	4 (9.3%)	2 (11.1%)	
	Chemotherapy	30 (69.8%)	11 (61.1%)	
	Chemotherapy+Anti-PD-1 Immunotherapy	9 (20.9%)	5 (27.8%)	0.801

*P<0.05 was considered significant. Chi-square test was used in this table.
