# Supplementary material for: Identification of Malassezia globosa as a Gastric Fungus Associated with PD-L1 Expression and Overall Survival of Patients with Gastric Cancer
Source: J Immunol Res. 2022 Nov 9;2022:2430759. doi: 10.1155/2022/2430759 (PMC9669766; doi:10.1155/2022/2430759)
Supplement: Supplementary 6 — Supplementary Table 4: univariate and multivariable Cox regression analyses for OS. [file 2430759.f6.pdf]

**Supplementary.Table.4** Univariate and Multivariable Cox regression analysis for OS.

| Characteristics | Univariate analysis[#] | | Multivariate analysis | |
|---|---|---|---|---|
| | HR, 95% CI | P-value | HR, 95%CI | P-value |
| Age (≥65 vs <65, years) | 2.311 (0.860-6.206) | 0.097 | - | |
| Gender (Male vs Female) | 0.621 (0.226-1.710) | 0.356 | - | |
| BMI (⩾24 vs <24, kg/m2) | 1.327 (0.460-3.823) | 0.601 | - | |
| Tumor Max Size (⩾4.6 vs <4.6, cm) | 1.711 (0.622-4.707) | 0.299 | - | |
| Tumor Location | | | | |
| Upper | 1 (Ref.) | | | |
| Middle | 0.426 (0.088-2.049) | 0.426 | - | |
| Lower | 0.631 (0.221-1.801) | 0.390 | - | |
| Tumor Differentiation (Poor vs High/Moderate) | 3.354 (0.729-15.437) | 0.120 | | |
| Lauren Classification | | | | |
| Intestinal | 1 (Ref.) | | | |
| Diffuse | 3.912 (0.887-17.255) | 0.072 | - | |
| Mix | - | - | | |
| pTNM Stage (IV vs I/II/III) | 5.801 (2.157-15.599) | **< 0.001** | 6.213 (2.275-16.966) | **< 0.001** |
| Lymphatic Vessel Invasive (Positive vs Negative) | 0.943 (0.328-2.716) | 0.914 | - | |
| Vascular Invasive (Positive vs Negative) | 1.925 (0.699-5.300) | 0.205 | - | |
| Nerve Invasive (Positive vs Negative) | 0.974 (0.891-1.066) | 0.572 | - | |
| HER2 Status (3+ vs 0/1+/2+) | 0.932 (0.212-4.105) | 0.926 | - | |
| PD-L1 Status (High vs Low) | 1.645 (0.617-4.384) | 0.320 | - | |
| *Malassezia globosa* (High vs Low) | 2.830 (1.061-7.554) | **0.038** | 3.080 (1.140-8.323) | **0.027** |
| Postoperative Treatment | | | | |
| None | - | - | | |
| Chemotherapy | 0.491 (0.178-1.351) | 0.168 | - | |
| Chemotherapy+Anti-PD-1 Immunotherapy | 1 (Ref.) | | | |

[#] The variables with P<0.05 were included in multivariate analysis

*P<0.05 was considered significant. HR: Hazard Ratio, CI: Confidence Interval.
